# Tannerella forsythia, Fusobacterium nucleatum, and Suspected Actinomyces Causing Massive Empyema: A Case Report

**DOI:** 10.7759/cureus.48117

**Published:** 2023-11-01

**Authors:** Courtney E Stewart, Allison McCafferty, Robert Sherertz

**Affiliations:** 1 Department of Internal Medicine, Grand Strand Medical Center, Myrtle Beach, USA; 2 Internal Medicine, Edward Via College of Osteopathic Medicine-Carolinas Campus, Spartanburg, USA

**Keywords:** anaerobes, actinomyces, fusobacterium nucleatum, tannerella forsythia, empyema

## Abstract

This report presents the case of a polymicrobial empyema due to *Fusobacterium nucleatum,* *Tannerella forsythia*, and suspected *Actinomyces* spp., presenting as several weeks of progressive shortness of breath and malaise. The patient had many risk factors for a lower respiratory tract infection, including chronic alcohol abuse, a flu-like illness months prior, and a recent invasive dental procedure. An admission CT scan showed a large right pleural effusion. Blood cultures were negative, but an aspirate from the pleural effusion showed abundant gram-positive rods that did not grow aerobically. Subsequent anaerobic cultures at a reference laboratory grew *Tannerella forsythia *and *Fusobacterium nucleatum*. This report will review the diagnostic difficulties associated with anaerobic causes of empyema in general and the specific organisms implicated in this case.

## Introduction

Pleural empyema is associated with high morbidity and mortality, and incidence is on the rise [1,2]. Medical literature classically reports that pleural empyema most commonly develops secondary to community-acquired pneumonia and also via contamination from adjacent anatomic structures. Early reports suggested empyemas were caused primarily by aerobic pathogens (*S. pneumoniae, H. influenzae,* and *S. aureus* [3-5]), but improved culture methodologies have demonstrated that anaerobic bacteria play an important role [3].

## Case presentation

A 60-year-old male presented to the emergency department (ED) complaining of a non-productive cough, dyspnea, and decreased appetite. He stated it took him an hour and a half to get dressed that morning secondary to significant shortness of breath, which prompted his ED visit. His dyspnea began 10 days prior, accompanied by chest heaviness, intermittent right upper quadrant pain, and an estimated 60-pound weight loss over the previous month. He denied fever, chills, or chest pain. Medical history was significant for hypertension, a mood disorder, and dental surgery six weeks prior. He had no history of cancer, immunosuppression, or recent steroid therapy. Social history was significant for a 15-pack-year smoking history, with cessation 15 years ago, alcohol use, and no known history of or exposure to tuberculosis.

Initial evaluation in the ED showed he was afebrile, tachycardic, mildly hypotensive, and tachypneic with an oxygen saturation of 95-97% on room air. The patient had poor dentition and decreased breath sounds at the right lung base. Initial laboratory results revealed a white blood cell (WBC) count of 32.2 x 10^9^/L and a ferritin of 997 ng/mL. Kidney and liver function tests remained normal throughout hospitalization.

**Figure 1:**
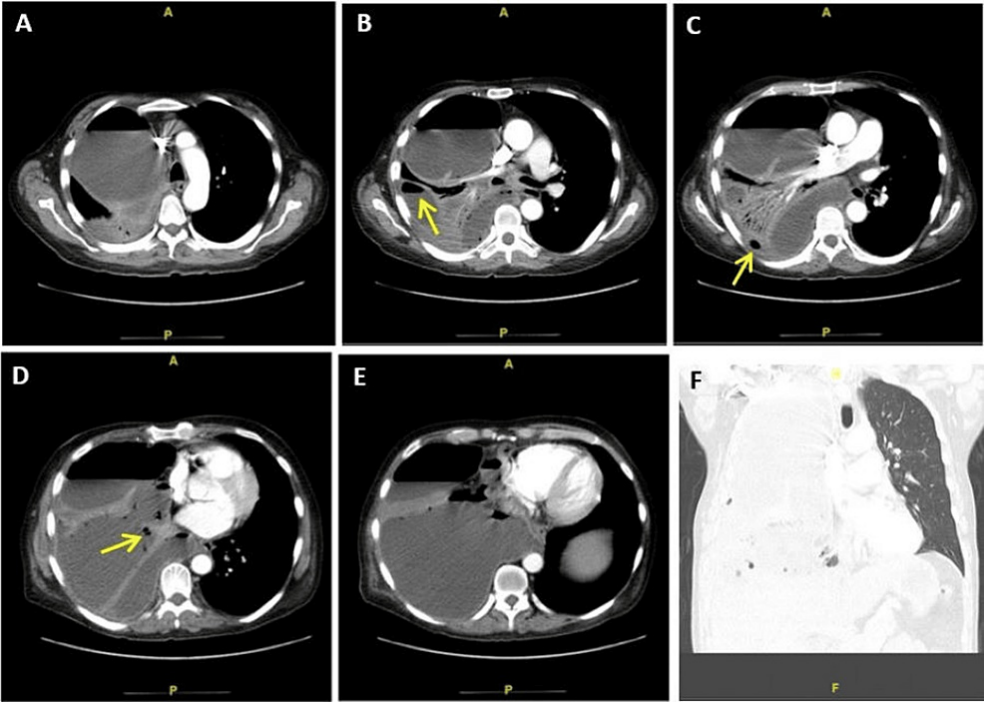
Images from CT thorax with contrast Images A to E: Anteroposterior (AP) view; Image F: Coronal view Note the air pockets within the fluid collection

Blood cultures were drawn and he received 2 g ceftriaxone and a 30 cc/kg intravenous (IV) fluid bolus for sepsis presumed secondary to community-acquired pneumonia. Subsequent imaging of the chest and abdomen revealed a massive right-sided pleural effusion, with gas components suggesting a possible empyema (Figures 1A-1F), prompting the addition of vancomycin and piperacillin-tazobactam for broader coverage, including anaerobes.

The patient was admitted for sepsis secondary to empyema. Interventional radiology performed thoracostomy placement, with the initial removal of 600 mL of pus-like material. Pleural fluid gram stain showed moderate WBCs and no organisms. Radiology recommended a barium swallow, which ruled out bronchopleural and esophagopleural fistula as potential sources.

**Figure 2:**
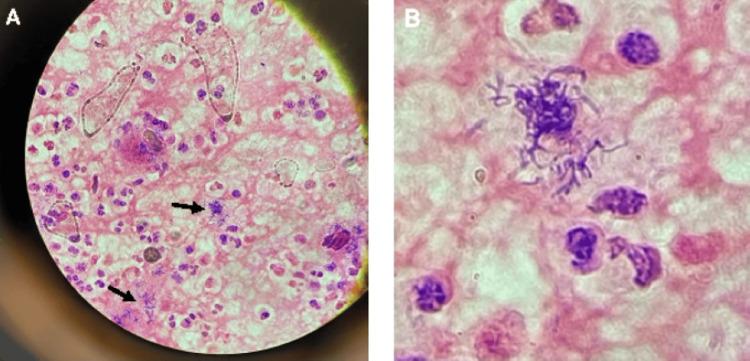
Histopathology images A. Gram stain of pleural fluid obtained during video-assisted thoracoscopic surgery (VATS) procedure. Arrows pointing at clumps of gram-positive rods. B. Gram-positive rods from A, magnified.

On day two of admission, cardiothoracic surgery performed a video-assisted thoracoscopic surgery (VATS) decortication where 3.5 L of pus-like fluid was removed with full re-expansion of the lung. Two chest tubes were placed. Antibiotics were changed to vancomycin, cefepime, and clindamycin. Pleural fluid collected during VATS was sent for cultures, fluid analysis, and cytology. Standard aerobic fluid cultures did not grow any bacteria, but the gram stain showed abundant gram-positive bacilli consistent in appearance with *Actinomyces* spp vs *Nocardia* (Figures 2A-2B). Additional acid-fast and anaerobic cultures were ordered, to be held for six weeks. An infectious disease specialist was consulted and recommended ampicillin-sulbactam monotherapy, which was continued throughout the hospitalization. 

Overall, the patient was hospitalized for eight days, before leaving against medical advice to care for a pet at home. On the day of discharge, he reported feeling much better, but still experiencing mild exertional dyspnea. Chest X-ray on the day of discharge showed significant improvement from admission but still displayed persistent right basilar lung and pleural disease with loculated pneumothoraces. All cultures, pathology, and cytology studies were negative through the date of discharge, so all antibiotic choices were made using clinical judgment. Based on Figures 2A-2B gram stain and suspected *Actinomyces* infection, the patient was discharged on amoxicillin, 1000 mg thrice a day (TID) for 30 days. Six weeks after discharge, anaerobic cultures had grown two anaerobes: *Tannerella forsythia *and *Fusobacterium nucleatum. *Unfortunately, the patient did not go to an outpatient follow-up appointment and was hospitalized just over a month later for dyspnea. Chest CT at that time showed decreased size of the empyema and improved aeration of the right middle and upper lung lobes. During the second hospitalization, the infectious disease specialist recommended amoxicillin-clavulanic acid 875 mg twice daily for three weeks on discharge.

## Discussion

Empyema is a challenging infection to diagnose and treat, likely contributing to its high morbidity and mortality. Delayed diagnosis can cause improper treatment, increasing the risk of complications: sepsis, respiratory failure, and lung abscesses. Empyema treatment typically involves a combination of antibiotics, drainage of the infected fluid, and supportive care. Antibiotic resistance can be problematic, especially in hospital-acquired infections [4].

We believe one of the most critical factors contributing to mortality in empyema is difficulty in identifying the causative agent, especially anaerobic organisms. A study that compared conventional microbiology with 16S ribosome gene amplification in 434 pleural space infections illustrated some of the diagnostic difficulties. They found that 35% of cases identified organisms by both standard cultures and nucleic acid amplification test (NAAT), 26% by standard cultures alone, 13% by NAAT alone, and 26% did not identify organisms by either methodology. If patients received antibiotics prior to pleural fluid analysis, 61% were culture-negative. In this study, if the *Streptococcus intermedius-anginosus-constellatus* group (which can grow anaerobically) was included with the anaerobes, then 41% of the isolates could be thought of as growing anaerobically [3].

*Fusobacterium nucleatum* is a gram-negative, spindle-shaped, anaerobe commonly found in the oral cavity, and also gastrointestinal and female genital tracts. *F. nucleatum* is an opportunistic pathogen associated with periodontitis, gingivitis, appendicitis, and various cancers. The bacterium possesses several virulence factors, including adhesins, lipopolysaccharides, and proteases, which allow it to adhere to host cells and evade its immune system [6,7]. *Tannerella forsythia* is another gram-negative anaerobe of the oral flora with similar characteristics to* F. nucleatum*. These bacteria can act synergistically to form complex biofilms that are difficult to treat [8,9].

While not confirmed by culture, we strongly suspect *Actinomyces* to have also been present based on the appearance of the pleural fluid gram stain that showed branching, gram-positive bacilli. *Actinomyces* is a group of gram-positive, anaerobic bacteria commonly found in the oral cavity and gastrointestinal and female genital tracts. While most species of *Actinomyces* do not cause disease, some can cause serious infections, particularly in immunocompromised individuals. *Actinomyces* has a filamentous growth pattern, resembling the hyphae of fungi. Its branching filaments aggregate into "sulfur granules" that can be seen microscopically. The Figure 2 gram stain shows organisms illustrating these characteristics. *Actinomyces* can also form biofilms. Risk factors for *Actinomyces* infections include alcohol abuse, poor dental hygiene, and oral tissue damage, such as dental procedures [10,11]. It is very likely this organism did not grow on cultures due to antibiotics already received or exposure to air associated with the prolonged time between initial specimen procurement and final inoculation in the reference laboratory.

Our patient had two risk factors clearly predisposing to anaerobic lung infections. Alcohol use increases the risk of aspiration [12]. The patient’s dental procedure is unknown, but likely related to his poor dentition, which is another risk factor [13]. The oral microflora associated with poor dentition is highly diverse, and most commonly includes *Porphyromonas gingivalis*, *Prevotella* *intermedia*, *Fusobacterium* *nucleatum*, *Actinomyces*, and *Bacteroides*. These are predominantly gram-negative organisms that are a mixture of aerobes and anaerobes [14]. Since most clinical microbiology laboratories do not routinely culture for anaerobic bacteria, anaerobes are often missed in empyemas.

A proposed solution to the difficulty in identifying the causative organism in empyemas is next-generation sequencing [15], which allows for more rapid identification. Another recent development in rapidly identifying anaerobes is matrix-assisted laser desorption-ionization time of flight mass spectrometry (MALDI-TOF MS/MALDI). Our institution has this technology but is awaiting verification by a reference lab. A meta-analysis comparing matrix-assisted laser desorption-ionization (MALDI) to 16S rRNA gene sequencing in identifying >6,500 strains of anaerobes found MALDI to be 92% accurate at determining genus and 84% accurate at determining species. While not as accurate as gene sequencing, MALDI’s speed and cost are more favorable. Proposed limitations of MALDI include a lack of anaerobic strains in the respective databases used [16-18]. Continued expansion of susceptibility testing for anaerobic databases would likely enhance MALDI’s accuracy. If a clinician's laboratory cannot perform anaerobic cultures, then the clinician must use clinical risk factors to guide their suspicion of anaerobic infection.

## Conclusions

In summary, the mortality of empyema has many contributing factors. Most important is the difficulty in isolating and identifying causative organisms. This is especially true if the bacterial etiology includes anaerobes. It is important to remember the potential role of anaerobes in empyemas to prevent delayed or misdiagnoses leading to improper treatment with its associated increased mortality and morbidity.
